# Synthesizing Metal Oxide Semiconductors on Doped Si/SiO_2_ Flexible Fiber Substrates for Wearable Gas Sensing

**DOI:** 10.34133/research.0100

**Published:** 2023-03-30

**Authors:** Feng Niu, Fugong Zhou, Zhixun Wang, Lei Wei, Jie Hu, Lei Dong, Yifei Ma, Mei Wang, Suotang Jia, Xuyuan Chen, Zhaomin Tong

**Affiliations:** ^1^ State Key Laboratory of Quantum Optics and Quantum Optics Devices, Institute of Laser Spectroscopy, Shanxi University, Taiyuan 030006, Shanxi, China.; ^2^ Collaborative Innovation Center of Extreme Optics, Shanxi University, Taiyuan 030006, Shanxi, China.; ^3^ School of Electrical and Electronic Engineering, Nanyang Technological University, Singapore 639798, Singapore.; ^4^ Center of Nano Energy and Devices, College of Information and Computer, Taiyuan University of Technology, Taiyuan 030024, Shanxi, China.; ^5^ Department of Microsystems, University of South-Eastern Norway, BorreN-3184, Norway.

## Abstract

Traditional metal oxide semiconductor (MOS) gas sensors have limited applications in wearable devices owing to their inflexibility and high-power consumption by substantial heat loss. To overcome these limitations, we prepared doped Si/SiO_2_ flexible fibers by a thermal drawing method as substrates to fabricate MOS gas sensors. A methane (CH_4_) gas sensor was demonstrated by subsequently in situ synthesizing Co-doped ZnO nanorods on the fiber surface. The doped Si core acted as the heating source through Joule heating, which conducted heat to the sensing material with reduced heat loss; the SiO_2_ cladding was an insulating substrate. The gas sensor was integrated into a miner cloth as a wearable device, and the concentration change of CH_4_ was monitored in real time through different colored light-emitting diodes. Our study demonstrated the feasibility of using doped Si/SiO_2_ fibers as the substrates to fabricate wearable MOS gas sensors, where the sensors have substantial advantages over tradition sensors in flexibility, heat utilization, etc.

## Introduction

With the development of industrialization and modernization, real-time detection of harmful and explosive gases is a long-term goal in safety control and environmental monitoring. Mine gas is a mixture of gases, mainly the explosive gas methane (CH_4_), produced during coal mining. A mine gas explosion may occur when the volume concentration of CH_4_ is in the 4.9% to 15.4% range [1]. Therefore, the continuous monitoring of CH_4_ in coal mines is highly demanded to ensuring a safe working environment. Currently, CH_4_ monitoring techniques used in coal mines are primarily accomplished by installing several gas sensors at fixed intervals. This technology has a few disadvantages, such as complex installation and fixed detection distances. Handheld gas sensors are an alternative means to measure CH_4_ around miners. However, handheld gas sensors have inherent limitations, such as large volumes, and are inconvenient to carry. Therefore, it is required to develop wearable gas sensors that can be easily carried to detect CH_4_ around miners in real time.

**Fig. 1.**
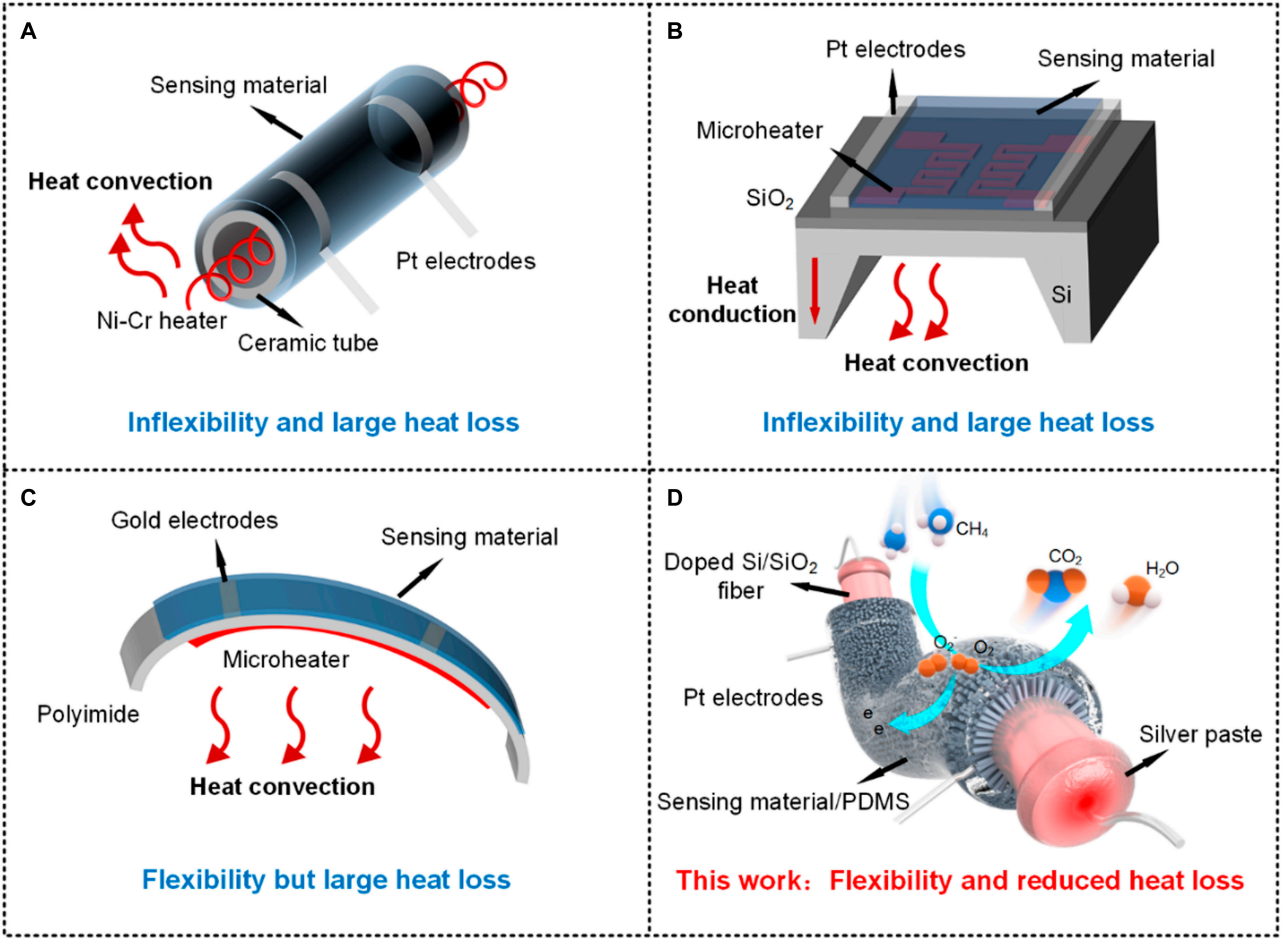
MOS gas sensors on different substrates. (A) Ceramic tube, (B) MEMS, (C) polyimide, and (D) doped Si/SiO_2_ fibers.

According to the working mechanism of gas sensors, they can be categorized as electrochemical, metal oxide semiconductor (MOS), catalytic combustion, optical, and acoustic. MOS gas sensors have attracted considerable attention because they are inexpensive, lightweight, and robust with remarkable sensing performances [2]. However, MOS gas sensors face great challenges in wearable application owing to extensive heat loss and inflexibility. As shown in Fig. 1A, the synthesized MOS nanomaterials were coated on ceramic tube substrates, forming sensing films after high-temperature calcination. A Ni-Cr heating wire was inserted into the ceramic tube to provide the high temperature required for the gas sensor [3,4]. However, these sensors have some disadvantages, such as large size, extensive heat loss, and inflexibility. To reduce the size and heat loss of the sensor, microelectromechanical systems (MEMS) have been used as alternate substrates (Fig. 1B). A microheater was fabricated to heat the sensing layer on the surface of the silica substrate by conventional lithography and nickel-etching techniques. Then, silica passivation layers and MOS nanomaterials were deposited for gas detection [5,6]. Despite that, the heat loss remained substantial owing to the thermal convection between the MEMS sensor and air and thermal conduction with the polysilicon substrate. In addition, MEMS sensors are not flexible, making them unsuitable for application to wearable devices. To make the sensor more flexible for application in wearable devices, polyimides have been used as substrates and inkjet-printed electrodes as heating sources [7,8], as shown in Fig. 1C. However, the performance of the sensor is poor owing to heat loss by thermal convection, an unstable heating temperature, and an uneven heating area.

As one-dimensional flexible substrates, fibers are widely used in wearable devices owing to their lightweight, high length-to-diameter ratio, excellent deformation ability, and the capability to be woven into fabrics [9,10]. The common methods for fabricating fibers include solution-based methods, dry fabrication, spinning processes, and thermal drawing. However, dry fabrication (e.g., physical [11] and chemical vapor depositions [12,13]) requires a vacuum environment and high temperatures. Solution-based methods (e.g., dip coating [14,15] and printing [16,17]) and spinning processes (e.g., electrospinning [18,19] and wet-spinning [20,21]) are difficult to scale up and unsuitable for complex architectures. By contrast, the thermal drawing method, a typical approach for industrially producing optical fibers, is advantageous for large-scale manufacturing and its feasibility for producing customizable structures [22,23]. Preforms with different structures are heated in a furnace and drawn into kilometers of microscopic fibers with fine internal structures. The viscous force, internal stress, and surface tension of the preforms are controlled to modulate the fine internal structures of the fibers [24]. The fibers can also be functionalized by integrating different materials (e.g., bulk polymers, glasses, metals, semiconductors, and nanomaterials) into the preform [22,25]. These composite fibers with functional structures have exhibited excellent potential in various research applications, including biosensors [26], supercapacitors [27], batteries [28], piezo/triboelectric generators [29], solar cells [30], and light-emitting devices [31].

In this study, we prepared a doped Si/SiO_2_ fiber substrate by the thermal drawing method, wherein the core was p-type silicon doped with boron and the cladding was silica. The diameters of the fiber and the core were 120 and 14 μm, respectively. The fiber was flexible, and the Joule heat generated by the core was conducted to the SiO_2_ surface with reduced heat loss. The SiO_2_ cladding acted as an insulating substrate compatible with conventional semiconductor manufacturing processes. Furthermore, dense and uniform Co-doped ZnO nanorods (NRs) with a length of 2 μm and a diameter of 200 nm were grown on the fiber surface through a hydrothermal method. Finally, a 30-μm-thick polydimethylsiloxane (PDMS) protective layer was applied on the fiber surface by dip coating. A schematic diagram of the sensor is shown in Fig. 1D. The sensor achieved a maximum response of 16% to 1,000 parts per million (ppm) CH_4_ at 50 °C, where the drive current of the sensor was 2.5 mA, and the power consumption of the sensor was 3.2 mW/mm. The response and recovery times of the sensor were approximately 350 and 106 s, respectively. Moreover, the sensor demonstrated excellent stability and durability under 10,000 bending cycles, various bending angles, and humidity conditions, indicating that doped Si/SiO_2_ fibers are ideal substrates for the fabrication of wearable MOS gas sensors.

## Results

### Fabrication of doped Si/SiO_2_ fibers

**Fig. 2.**
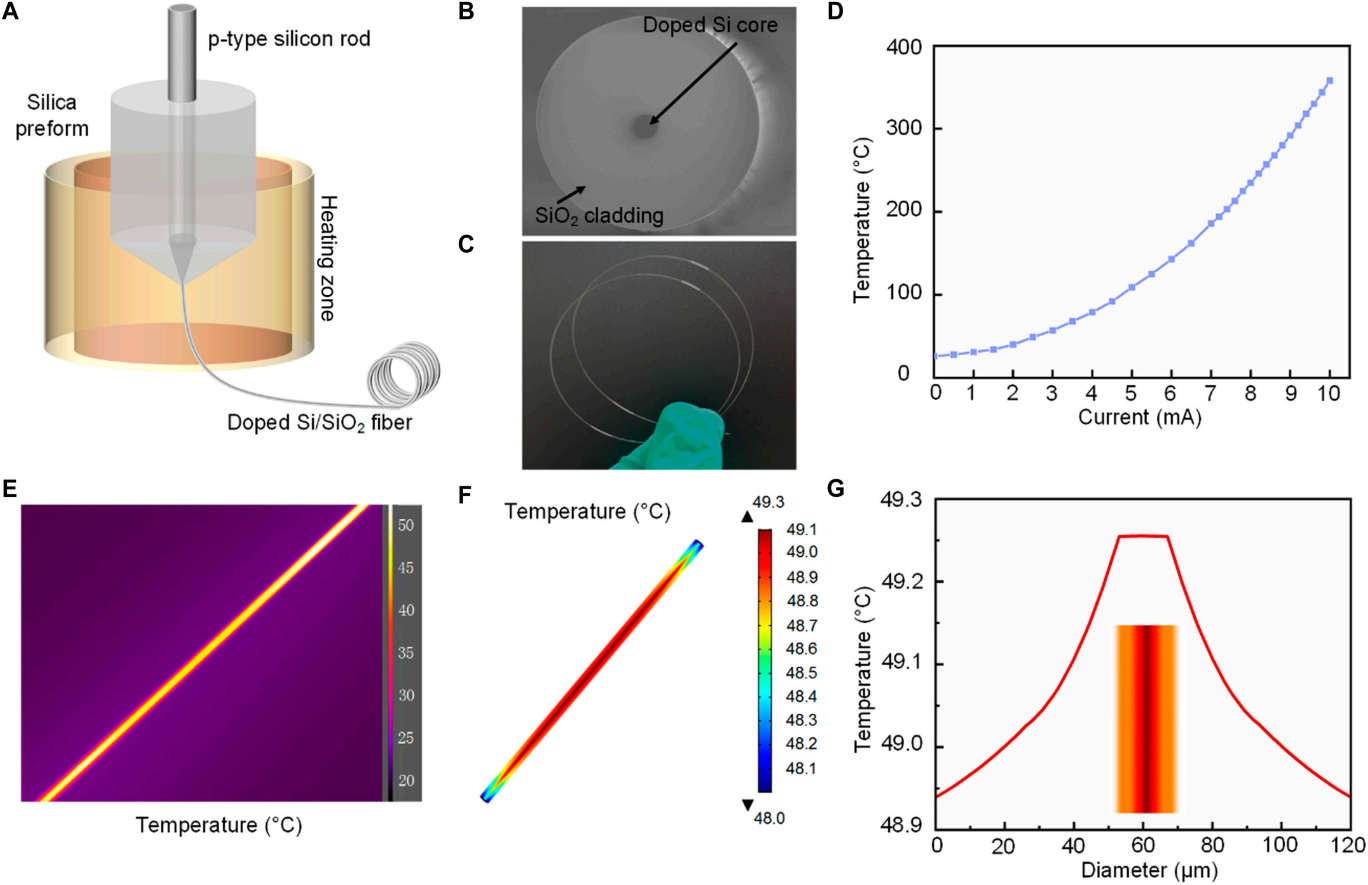
Fabrication and characterization of fibers. (A) Schematic of the thermal drawing process to prepare doped Si/SiO_2_ fibers. (B) Cross-sectional SEM image of the doped Si/SiO_2_ fibers. (C) Optical image of the resulting flexible fiber. (D) Variation in the temperature of the fiber surface with current. (E) Thermal image of doped Si/SiO_2_ fibers at 50 °C. (F) Simulation results of fiber temperature distribution. (G) Variation of the simulated fiber temperature across its diameter.

The approach to producing doped Si/SiO_2_ fibers is illustrated in Fig. 2A. First, a heavily doped p-type silicon rod was inserted into a thick-walled silica tube closed at one end to prepare a preform that was then fixed in a vertical tube furnace at a temperature of 2,000 °C. After approximately half an hour, the silica cladding material in the heating zone softened. To draw a continuous fiber, the preform was fed into the heating zone at a constant speed, while the fiber was drawn from the bottom and collected by a capstan roller at a higher speed. For devices in the fiber form factor, a thinner diameter offers better flexibility. However, an extremely thin fiber device can be very difficult to handle and weave. To balance the flexibility and processability, we picked 120 μm as the outer diameter, which was close to the size of thick human hair. The resulting fiber, in the selected sizes, was flexible yet feasible for further handling and being woven into a fabric. In addition, the doped Si core was used as the Joule heating component for the sensor. Thus, a continuous Si core should be achieved. To ensure this, the core size had a lower limit to avoid capillary instability, which might cause discontinuity in the fiber core during the thermal fiber drawing. A size larger than 10 μm was in a safe region to avoid capillary instability. Also, a large cladding-to-core ratio was good for maintaining the concentric geometry of the core-clad structure and promoted an even heating of the active materials grown on the outer surface. A thick Si core might decrease the mechanical strength of the fiber device, since Si was brittle and fragile. Therefore, we picked 14 μm as the Si core diameter, considering the machinability of Si. A cross-sectional image of the fabricated fiber is shown in Fig. 2B. The fiber and core diameters were 120 and 14 μm, respectively. The resistivity of the core was 0.02 Ω·cm. As shown in Fig. 2C, the resulting fiber was very flexible. After the silver paste was applied and sintered at both ends of the doped Si, an ohmic contact was achieved. Joule heating was generated by applying a current across the doped Si, and the heat was transferred to the fiber surface through the SiO_2_ cladding by heat conduction. The resulting temperature on the fiber surface depended on the applied current (Fig. 2D); a thermal image of the fiber surface under 2.5 mA current is shown in Fig. 2E.

To further investigate the temperature gradient inside the fiber, a finite-element simulation was performed using a combination of the solid heat transfer and current modules in COMSOL Multiphysics software. All the simulation parameters and geometries of the doped Si/SiO_2_ fibers were defined according to the actual materials and structure. Moreover, the same boundary conditions used in the experiments were applied, and the simulation parameters are presented in Note S1. As shown in Fig. 2F, the Joule heat generated by the doped Si core was conducted to the surface of the fiber, and the temperature distribution was uniform on the entire surface. Only the surfaces near the ends had slightly lower temperatures owing to heat convection. The temperature distribution along the diameter is shown in Fig. 2G. The doped Si core temperature was approximately 49.3 °C, and the SiO_2_ surface temperature was approximately 48.9 °C, indicating that the heat loss by the heat conduction through the SiO_2_ layer is 32.7 mW (the calculation procedure is presented in Note S2). The fiber heating curves are shown in Fig. S1.

### Fabrication of Co-doped ZnO NRs gas sensors

**Fig. 3.**
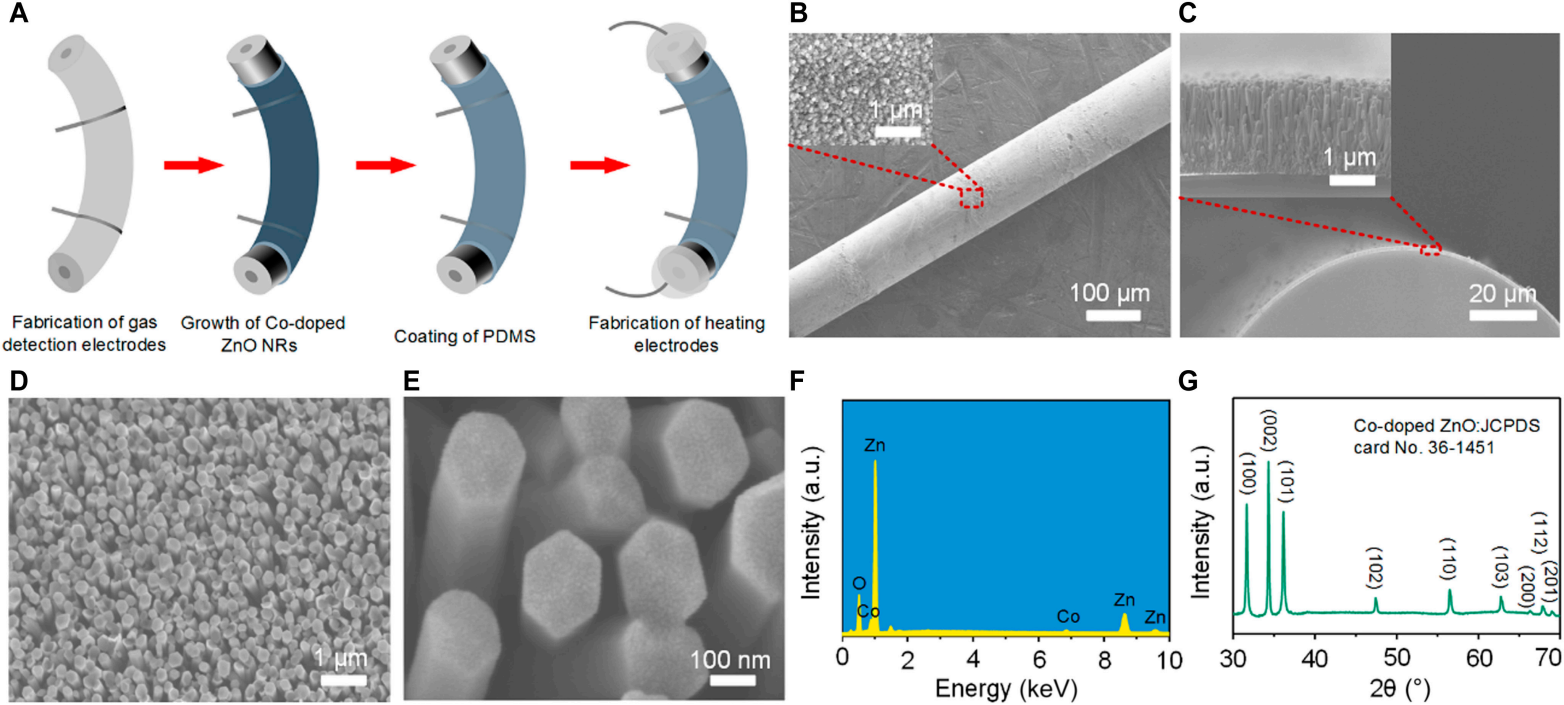
Fabrication of gas sensors. (A) Illustration of the fabrication process of Co-doped ZnO NRs gas sensors. (B) SEM image of the ZnO seed layers grown on the doped Si/SiO_2_ fibers; the inset is a magnified image of the selected area. (C) Cross-sectional SEM image of the Co-doped ZnO NRs grown on the doped Si/SiO_2_ fibers; the inset is a magnified image of the selected area. (D and E) SEM images of the surface of Co-doped ZnO NRs at different scales. (F) EDS spectrum of the Co-doped ZnO NRs. (G) XRD pattern of the Co-doped ZnO NRs.

As a MOS gas sensing material, ZnO has been widely used to detect dangerous and harmful gases because of its desirable properties, such as its wide band gap (3.37 eV), large exciton binding energy (60 meV), high mobility, and high chemical and thermal stability [32,33]. However, pure ZnO requires a high operating temperature (around 260 °C) to obtain the highest sensor response. The high operating temperature requires a high drive current of 9 mA for doped Si, which makes the drive voltage (68 V) exceed the safe limit for the human body and consumes a larger amount of power (30.7 mW/mm). The preparation process of pure ZnO NRs and the gas sensing test results are provided in Note S3. Many studies have reduced the operating temperature by doping ZnO with noble metals (e.g., Au [34], Ag [35], and Pd [36]) and transition metals (e.g., Cu [37], Ni [38], and Co [39]). Among them, the operating temperature of Co-doped ZnO is the lowest, which meets the requirements of safe limits of drive current and drive voltage for the human body in wearable sensing. Co-doping yields better gas sensing properties by reducing the operating temperature due to the increased active sites that facilitate and enhance the interaction with CH_4_ gas molecules. Therefore, Co-doped ZnO NRs were prepared on the surface of doped Si/SiO_2_ fibers. The preparation process of the gas sensor is shown in Fig. 3A. First, the fiber was cut into 2-cm-long pieces, then ultrasonically cleaned with acetone, ethanol, and deionized water for 15 min to remove organic contaminants and dust from the surface. Next, the fiber was treated in a vacuum plasma instrument for 90 s to activate the surface and increase its hydrophilicity. By sintering silver paste, platinum wires and the fiber were connected to form gas detection electrodes. Subsequently, seed layers consisting of ZnO nanoparticles were introduced by a sol-gel method by dip coating the fiber. The introduction of the ZnO seed layer could effectively reduce the crystal nucleation barrier for the subsequent coating and minimize the lattice mismatch; Co-doped ZnO NRs were grown in situ on the seed layer using a hydrothermal method. Co doping concentration influences sensor response, and the maximum sensor response is obtained when the Co doping concentration is 10 mM [39]. Therefore, Co-doped ZnO NRs were grown by adding 10 mM Co(NO_3_)_2_·6H_2_O into the hydrothermal solution to prepare pure ZnO NRs. The cross-sections of the fiber were immersed in a dilute hydrochloric acid solution to remove excess Co-doped ZnO NRs. When the Co-doped ZnO NRs on the fiber surface are exposed to air for a long time, the NRs may detach under external forces; water molecules in the air also occupy the active sites on the surface of the sensing materials, thereby influencing the adsorption and equilibrium of the target gas. PDMS is a common polymer widely used in smart wearable devices because of its low Young's modulus, excellent gas permeability, and good hydrophobicity [40,41]. There are many methods in preparing thin PDMS films, such as spin coating, drop coating, and dip coating. The spin coating process is not compatible with the one-dimensional shape of the fiber, and the PDMS film prepared by the drop coating method is not uniform. Therefore, we used the dip coating method to coat PDMS on the fiber surface. The thickness of the PDMS layer relates to the pulling speed of the fiber. When the withdrawal speed is 100 μm/s, the thickness reaches a minimum of 30 μm. Therefore, a 30-μm-thick PDMS protective layer was coated on the fiber surface using the dip coating method. The detailed preparation process is provided in Note S4. Finally, platinum wires were connected to both ends of the fiber by sintering silver paste to form heating electrodes.

The fiber surface was evenly covered by the ZnO seed layers consisting of nanoparticles as shown in Fig. 3B. Figure 3C shows a scanning electron microscopy (SEM) image of the cross-section of the fiber, wherein a dense and uniform layer of Co-doped ZnO NRs with an average length of 2 μm was observed (inset). The top view of the fiber surface (Fig. 3D) showed that the Co-doped ZnO NRs formed a dense interconnected random network with directional channels for gas diffusion. By this growth procedure, the diameters of the Co-doped ZnO NRs were approximately 200 nm, and the diameter distribution was relatively uniform. Figure 3E shows the quasi-hexagonal facets in the Co-doped ZnO NRs, consistent with the growth habit of wurtzite crystals. Energy-dispersive spectroscopy (EDS) of the Co-doped ZnO NRs (Fig. 3F) confirmed that they were composed of Zn, O, and Co. A quantitative EDS analysis of the elements is presented in Fig. S3. The x-ray diffraction (XRD) pattern of the Co-doped ZnO NRs (Fig. 3G) was in agreement with the hexagonal wurtzite ZnO crystal structure (JCPDS card No. 36-1451), and no additional diffraction peaks were observed. Given that the ionic radii of tetrahedrally coordinated Co^2+^ and Zn^2+^ are similar [42], the absence of the diffraction peaks of cobalt oxides in the XRD pattern implied that the Co was present in the ZnO matrix by substituting Zn and without changing the wurtzite structure of ZnO [43,44]. A strong peak at 34.3° can be related to the Co-doped ZnO (002) plane, indicating that the growth direction was along the *c*-axis of the Co-doped ZnO and normal to the substrate plane.

### Gas sensing measurements

The gas sensing properties were tested at a fixed relative humidity (RH) of 30 ± 5%, and a mass flow system was used to control the CH_4_ gas concentration. The gas mixture (N_2_ and CH_4_) was delivered into the gas chamber at a constant flow rate of 200 standard cubic centimeters per minute. A constant current source was connected to both ends of the heating electrode, and the sensor resistance was monitored using a source measurement unit under a constant DC bias voltage of +5 V. To study the gas sensing properties, the sensor sensitivity (*S*) is defined by the following equation:

$$S\left(\%\right)=\left[\left(R_{a}-R_{g}\right)/R_{a}\right]\times 100\%$$ (1)

where *R_a_* is the initial resistance in air and *R_g_* is the resistance in the target gas. The response time (*τ*_res_) and recovery time (*τ*_rec_) are defined as the times required for the sensor output to reach 90% of the total resistance change in the cases of adsorption and desorption, respectively.

### Gas sensing performance

**Fig. 4.**
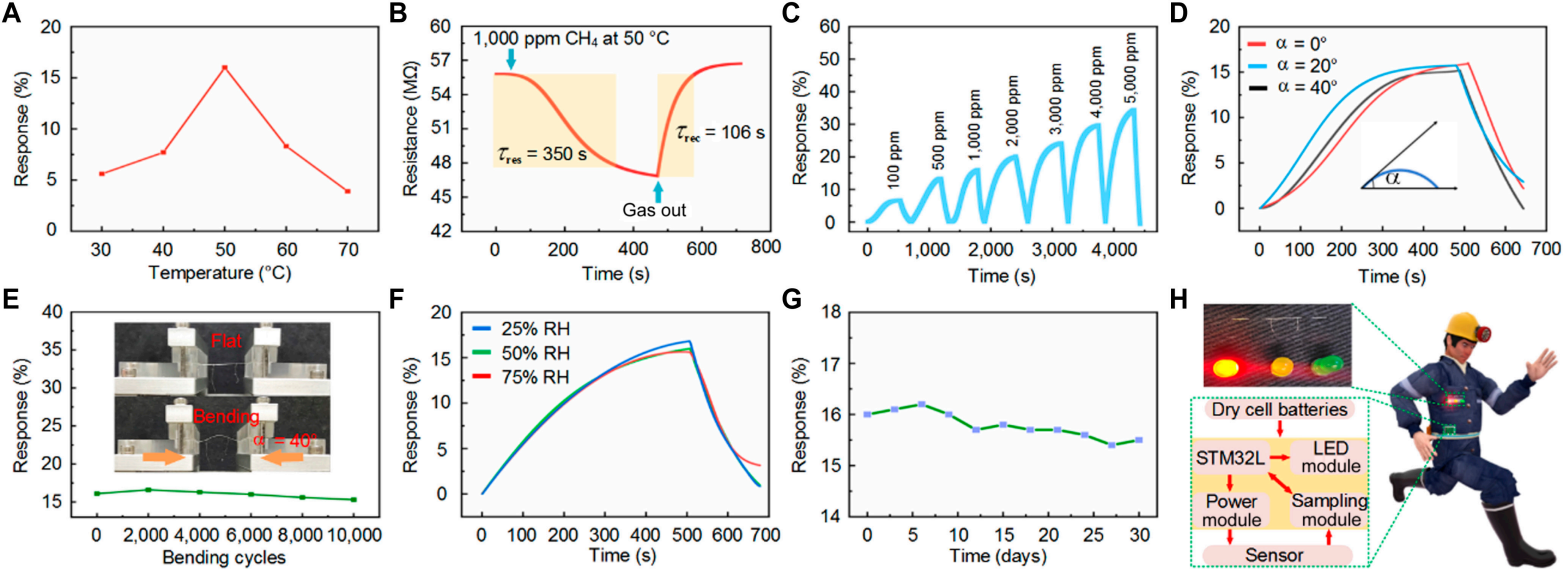
Fabrication and performance testing of the sensor. (A) Response of the sensor at various operating temperatures when exposed to 1,000 ppm CH_4_ gas. (B) Transient response–recovery curve of the sensor exposed to 1,000 ppm CH_4_ at 50 °C. (C) Dynamic response–recovery curve at different concentrations of CH_4_ at 50 °C. (D) Transient response–recovery curves of the sensor exposed to 1,000 ppm CH_4_ at various bending angles; the definition of the bending angle is provided in the inset. (E) Response of the sensor to 1,000 ppm CH_4_ after being bent multiple times at a bending angle of 40°; the inset shows one bending cycle in measurement. (F) Transient response–recovery curves of the sensor exposed to 1,000 ppm CH_4_ at different humidity levels. (G) Long-term stability of the sensor exposed to 1,000 ppm CH_4_ at 50 °C. (H) Schematic of the gas sensor; the inset is the magnified image of the selected area.

Figure 4A shows the response of the sensor to 1,000 ppm CH_4_ at different operating temperatures. The sensor response was observed to first increase and then decrease while increasing the temperature from 30 to 70 °C by changing the drive current. The maximum response (16%) was achieved at 50 °C, where the drive current and power consumption of the sensor were 2.5 mA and 3.2 mW/mm, respectively. This could be explained by the temperature-dependent gas adsorption and desorption behaviors on the surface of the sensing material. When the temperature was below 50 °C, gas adsorption was faster than desorption. Thus, in this regime, the enhanced gas adsorption caused the response of the sensor to increase with temperature. At 50 °C, gas adsorption and desorption reached an equilibrium, resulting in maximum response. When the operating temperature was above 50 °C, gas desorption was faster than adsorption, leading to a continuous decrease in the response. Response and recovery characteristics are critical parameters for evaluating the sensing performance of gas sensors. Figure 4B presents the typical response–recovery curve at the optimum operating temperature (50 °C). When the sensor was exposed to 1,000 ppm CH_4_, the resistance decreased and stabilized with time. However, when air was introduced, the resistance increased and returned to nearly the initial value. As shown in Fig. 4B, the response time and recovery time were approximately 350 and 106 s, respectively. The response–recovery measurement results of the sensor without the PDMS protective layer are shown in Fig. S4 for 1,000 ppm CH_4_. Gas permeability is thickness-dependent for PDMS thicknesses smaller than some tens of micrometers [45]. The thicker the PDMS, the lower the permeability, and the lower the sensor response and the gas detection limit. As shown in Fig. S4B, the response of the sensor decreased by 2.4% after coating the 30-μm-thick PDMS protective layer. Figure 4C shows the dynamic response and recovery curves when detecting different concentrations of CH_4_ at 50 °C. As the CH_4_ concentration increased from 100 to 5,000 ppm, the response value gradually increased, demonstrating good sensing ability for different concentrations of CH_4_.

The sensor performance when mechanically deformed was studied by bending experiments. Figure 4D shows the dynamic response–recovery curves of the sensor when it was bent at angles of 0°, 20°, and 40° (exposed to 1,000 ppm CH_4_ at 50 °C). The corresponding responses were 16%, 15.7%, and 15.2%, respectively. A reduction in the response value was because the binding energy and charge transfer decreased between the sensing layer and the target gas molecules at a higher bending angle. As shown in the fatigue test response–recovery (Fig. 4E), the sensor still maintained a good response with minor deterioration (less than 5%) after 10,000 bending cycles (a bending angle of 40° in each cycle), indicating its excellent mechanical durability and robustness. Figure 4F shows the dynamic response–recovery curves under 25%, 50%, and 75% RH (exposed to 1,000 ppm CH_4_ at 50 °C). The sensor exhibited a stable response under high humidity, further strengthening its applicability in practical wearable applications. Figure 4G shows the long-term stability test of the sensor, wherein the time-dependent response values were recorded within 30 days for 1,000 ppm CH_4_. The sensor maintained 97.5% of the initial response value, confirming that the as-prepared sensor had good long-term stability for CH_4_ detection.

Considering the sensor in practical applications, we wove the sensor into a miner cloth and designed an alarm circuit board to connect the sensor, as shown in Fig. 4H. The circuit board was mainly composed of a power module, a resistance sampling module, an STM32L microprocessor, and a light-emitting diode (LED) alarm module. The dry cell batteries provided stable power for the circuit board. The low-power consumption STM32L microprocessor realized the function of data collection and processing. The power module provided a stable constant drive current of 2.5 mA to achieve the sensor operating temperature of 50 °C. The resistance sampling module monitored the resistance change of the sensor. Through preset resistance thresholds, red-, yellow-, and green-colored LEDs indicated the concentration ranges of CH_4_. When the concentration of CH_4_ exceeded 5,000 ppm (e.g., resistance lower than 37 MΩ), the red LED turned on to warn of the highest hazard level that is close to an explosion (Movie S1). Long-term exposure to a medium concentration of CH_4_ environment could cause critical damage to the human body. Such a situation could be indicated by the yellow LED, i.e., when the concentration of CH_4_ was 100 to 5,000 ppm (e.g., resistance between 54 and 37 MΩ). The green LED indicated a safe working environment, which remained on only when the concentration of CH_4_ was lower than 100 ppm (e.g., resistance higher than 54 MΩ).

## Discussion

In this study, doped Si/SiO_2_ flexible fibers were prepared by a thermal drawing method, and Co-doped ZnO NRs were in situ grown on the fiber surface to obtain a flexible wearable gas sensor to detect CH_4_ gas. The doped Si/SiO_2_ fibers were flexible, with reduced heat loss. The Joule heat generated by the doped Si fiber core was conducted to the SiO_2_ surface and provided the optimal temperature (50 °C) for Co-doped ZnO NRs to detect CH_4_ gas. The SiO_2_ cladding acted as an insulating substrate that is compatible with conventional semiconductor manufacturing processes. The flexible gas sensor exhibited excellent stability and durability in a high-humidity environment with more than 10,000 bending cycles and the power consumption of the sensor was 3.2 mW/mm. In addition, the fiber could maintain its excellent sensing performance at high temperatures (up to 1,400 °C), potentially enabling more material growth processes, and laying a solid foundation for further applications. Therefore, the feasibility of using doped Si/SiO_2_ fibers as the substrates to fabricate wearable MOS gas sensors was demonstrated and verified. The MOS gas sensors fabricated on doped Si/SiO_2_ fibers are superior to traditional sensors in terms of flexibility, heat utilization, etc., and thus they have promising application prospects in gas sensing.

## Materials and Methods

### Materials

Zinc acetate dihydrate (Zn(CH_3_COO)_2_·2H_2_O) and anhydrous ethanol were purchased from Aladdin Biochemistry Technology Co., Ltd. (Shanghai, China). Cobalt nitrate hexahydrate (Co(NO_3_)_2_·6H_2_O), zinc nitrate hexahydrate (Zn(NO_3_)_2_·6H_2_O), and hexamethylenetetramine (HMTA) were provided by Sinopharm Chemical Reagent Co., Ltd. (Shanghai, China). The conductive silver paste was obtained from Guangzhou Techno Electronic Technology Co., Ltd. (Guangzhou, China). PDMS prepolymer and curing agent were purchased from Dow Corning Chemical Co., Ltd. (America). All chemicals were of analytical grade and used without further purification.

### Fabrication of doped Si/SiO_2_ fibers

The fiber was fabricated by inserting a heavily doped p-type silicon rod into a thick-walled silica tube closed at one end and heating to 2,000 °C. After a period of heating, the fiber was drawn from the softened preform and necked to a controllable diameter under an external force by turning capstans. In the drawing process, the cladding material softened, and the core was in a flowable state. The thermal drawing process conforms to the principle of constant volume, and a detailed explanation is provided in Note S5. The preform was fed into a furnace at a rate of 0.5 mm/min and drawn at a speed of 10 m/min. Several meters of fibers were produced as the diameter of the preform was reduced from ~17 mm to 120 μm, and the core diameter was reduced from 2 mm to 14 μm. The average composition of the draw was consistent from one end to the other.

### Fabrication of the ZnO seed layer

Zn(CH_3_COO)_2_·2H_2_O (0.16 g, 30 mM) was dissolved in anhydrous ethanol while magnetically stirring it for an hour to yield a homogeneous solution. ZnO seed layers were deposited on the fiber substrates by dip coating with a withdrawal speed of 1 cm/min at room temperature. After coating, the fiber was dried at 180 °C in a heating coil for 5 min to evaporate the solvent from the seed layer. The dip coating and drying processes were repeated 5 times. Finally, the coating was calcined at 300 °C for 30 min to obtain ZnO seed layers.

### Growth of Co-doped ZnO NRs

Co-doped ZnO NR growth was performed using a hydrothermal method. Fibers with grown seed layers were placed vertically in an aqueous solution (20 ml) of Zn(NO_3_)_2_·6H_2_O (0.15 g, 25 mM), HMTA (0.07 g, 25 mM), and Co(NO_3_)_2_·6H_2_O (0.06 g, 10 mM) at 90 °C for 8 h in a beaker covered with a Petri dish to minimize evaporation. The fibers were then washed several times with deionized water to remove excess NRs on the surface. Eventually, Co-doped ZnO NRs were obtained on the surface of the fiber by drying at 60 °C for 6 h and annealing at 400 °C for 2 h.

### Characterization

SEM images were taken using a field-emission scanning electron microscope (Hitachi, SU8010). The XRD pattern was collected using a D8 Advance x-ray diffractometer (Empyrean) with Cu Kα radiation in the range 10° < 2*θ* < 80°. The compositions of the materials were examined using energy-dispersive spectroscopy (AMETEK, EDAX). Thermal images were captured using a thermal camera (FLIR, A655sc). The resistance of the sensor was characterized using a Keithley 2461 sourcemeter. The current was supplied by a Keithley 6220 precision current source. A mass flow control system (Zhongji, MF-3D) was used to modulate the CH_4_ concentration in the N_2_ gas. The mechanical properties were tested on an electronic flexibility testing machine (Prtronic, FT2000).

## Data Availability

All relevant data supporting the findings of this study are available within this article and its Supplementary Materials or from the corresponding author upon reasonable request.

## Supplementary Materials

Supplementary 1 Note S1. Parameters for the finite-element simulation. Note S2. Calculation process of sensor heat loss. Note S3. The preparation process of pure ZnO NRs and gas sensing test results. Note S4. The detailed preparation process of the PDMS coating layer. Note S5. Fabrication of doped Si/SiO_2_ fibers. Fig. S1. Simulation results of the fiber heating process. Fig. S2. Gas sensing test results of the sensor. Fig. S3. EDS mapping of Co-doped ZnO NRs. Fig. S4. Gas sensing test results of the sensor without a PDMS protective layer. References [46–48]
